# Physiology and Survival of Intertidal Calcifiers in Two Contrasting Upwelling Systems

**DOI:** 10.1002/ece3.73065

**Published:** 2026-02-10

**Authors:** Alejandro Hernández‐Dauval, Andrés Valenzuela‐Sánchez, Marco A. Lardies, Leonardo D. Bacigalupe

**Affiliations:** ^1^ Facultad de Ciencias, Instituto de Ciencias Ambientales y Evolutivas Universidad Austral de Chile Valdivia Chile; ^2^ Institute of Zoology, Zoological Society of London London UK; ^3^ Facultad de Artes Liberales Universidad Adolfo Ibáñez Santiago Chile; ^4^ Instituto Milenio de Socio‐Ecología Costera (SECOS) Santiago Chile

**Keywords:** climate change, heart rate, mortality, oxygen consumption, pH

## Abstract

Climate change alters the oceans' temperature, pH, and oxygen concentration. These changes are expected to increase globally over the coming decades, affecting a wide range of marine organisms. Coastal upwelling zones, characterized by their high environmental variability, serve as ideal natural laboratories to study the potential impacts on marine organisms and ecosystems of temperature change, acidification, and ocean deoxygenation. The estimation of survival using capture‐mark‐recapture (CMR) data has been commonly applied to vertebrates, and to date, very few studies have been done on marine invertebrate organisms. In this study, we combined field CMR data and laboratory measurements to assess the physiological responses (metabolic rate and heart rate) and survival probability of individuals in two populations of intertidal mollusks, *Chiton granosus* and *Scurria zebrina*, in contrasting upwelling environments (i.e., semi‐permanent vs. seasonal). We found that (1) there are no differences between the two studied populations for heart rate in both species, (2) the *S. zebrina* population subjected to seasonal upwelling has a higher metabolism, (3) there are no differences in the calcification rate between the two studied populations of both species, and (4) survival is significantly higher in the semi‐permanent upwelling location for both species. Our findings highlight species‐specific responses to contrasting upwelling regimes, suggesting that phenotypic plasticity and survival differences may influence resilience under ongoing climate change.

## Graphical abstract

This study evaluates the physiological responses and survival of two intertidal mollusk species, *Chiton granosus* and *Scurria zebrina*, across contrasting upwelling environments. We found that metabolic rates and survival differ between populations exposed to seasonal versus semi‐permanent upwelling. These findings highlight the importance of phenotypic plasticity and environmental variability in shaping species' resilience to climate change in coastal ecosystems.

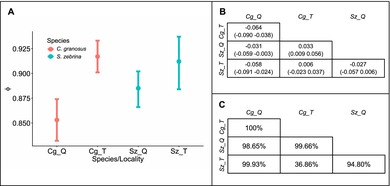

**Abbreviations**
CMR: capture–mark–recapture
DIC: Dissolved inorganic carbon
pHT: pH on the total scale
SMR: standard metabolic rate
ST: sea surface temperature
TA: total alkalinity
Ω: carbonate saturation state (calcite or aragonite)

## 1 Introduction

The oceans are significantly impacted by global climate change as they absorb heat and greenhouse gases from the atmosphere, a process that drives ocean warming, acidification, and deoxygenation (IPCC 2023). These changes disrupt key environmental parameters (e.g., temperature, pH, and oxygen levels), threatening marine life and generating socio‐economic challenges, such as risks to food security and fisheries sustainability (Gawel et al. 2023; Pimentel et al. 2023).

Coastal upwelling regions, where cold, nutrient‐rich subsurface waters rise to the surface, are among the most vulnerable ecosystems to these combined stressors (low temperatures, ocean acidification, and deoxygenation) (Gruber 2011). These regions are characterized by low temperature, oxygen, pH, and calcium carbonate saturation states (Ω), as well as high CO_2_ partial pressure (*p*CO_2_) and dissolved inorganic carbon (DIC) (Torres et al. 2011; Vargas et al. 2017). The impact of climate change on upwelling ecosystems is a subject of considerable scientific interest (Bograd et al. 2023), as it is predicted that wind intensification and ocean warming will alter the seasonality and duration of upwelling in the coming decades (Du et al. 2024; Fernández et al. 2024).

Organisms in these zones often exhibit traits similar to those observed under experimental acidified conditions (Mekkes et al. 2021), suggesting a preexisting adaptation to stressful conditions. Recent studies have provided mechanistic insights into how organisms from upwelling systems cope with extreme environmental variability. Experimental work on intertidal invertebrates shows that chronic exposure to fluctuating pH, temperature, and oxygen levels enhances physiological plasticity, including adjustments in metabolic rates, acid–base regulation, and cellular stress responses (Fernández et al. 2024; Dong 2023). Meta‐analyses show that, while bivalves typically depress metabolism under low pH, populations from strong upwelling regions tend to maintain or even increase metabolic activity, indicating reduced sensitivity to acidified conditions (Czaja et al. 2023).

Consequently, as upwelling zones resemble ocean conditions that will be more common in other areas as climate change progresses, these areas serve as natural laboratories to study the effects of global change drivers as forces of natural selection in marine environments (Gaitán‐Espitia et al. 2017; Pespeni et al. 2013; Vargas et al. 2017). The study of these populations allows for a better understanding of phenotypic plasticity and physiological tolerance ranges, which are crucial for predicting which species might withstand the future upwelling intensification on the Southern Eastern Pacific coast (Fernández et al. 2024).

In northern Chile, year‐round upwelling creates semi‐permanent upwelling hotspots, such as in the coastal area of Talcaruca (Figueroa and Moffat 2000), where current pH levels are comparable to those projected for other oceanic regions by 2100 (Gruber 2011; Torres et al. 2011; Vargas et al. 2017). These conditions shape rocky shore communities and act as biogeographic barriers, limiting species distribution (Broitman et al. 2018, 2021). In contrast, seasonal upwelling areas, like Quintay Bay, exhibit an intermittent seasonal frequency (Broitman et al. 2001; Pulgar et al. 2011; Pérez‐Matus et al. 2017) that has a less pronounced impact on intertidal and subtidal communities (Broitman et al. 2001). Such contrasting abiotic conditions provide a unique opportunity to study how environmental variability influences survival rates and physiological traits within populations of the same species.

**FIGURE 1:**
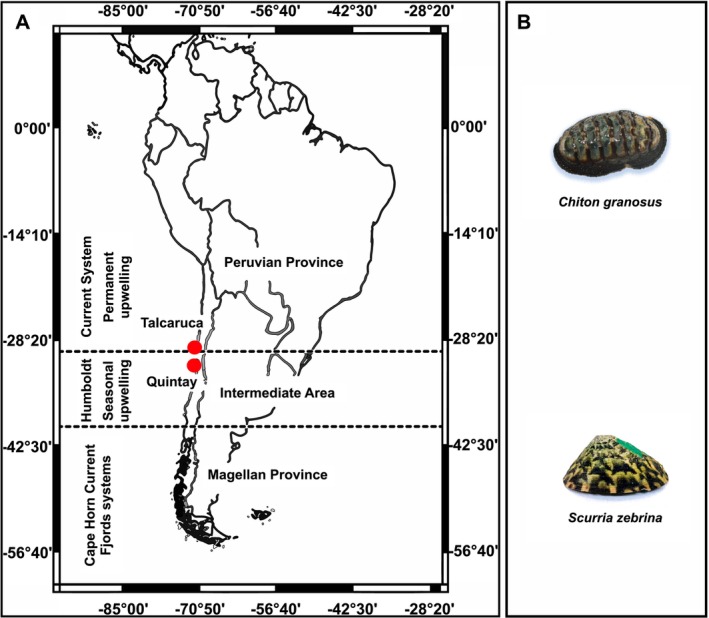
(A) Map of the South Pacific showing the localities examined in this study (Talcaruca and Quintay). The dotted lines separate the biogeographic provinces along the coast of Peru and Chile, indicating different differential upwelling systems. (B) Studied intertidal mollusk species.

Mollusks, particularly sensitive to pH and temperature changes, are ideal indicators of these impacts (Gattuso et al. 2015). Prolonged exposure to low pH can compromise shell integrity (Beniash et al. 2010; Avignon et al. 2020) and induce metabolic depression as a strategy that reduces mortality risk (Guppy and Withers 1999; Navarro et al. 2013, 2016). However, species from upwelling zones may exhibit local adaptation to low pH levels (Hofmann et al. 2014; Czaja et al. 2023) as seen in enhanced physiological performance in some chiton species (Manríquez et al. 2021). This study focuses on *Scurria zebrina* (Lesson 1830) and *Chiton granosus* (Freembly, 1827) (Figure 1B), two abundant mollusk species along the southeastern Pacific coast (Fernández et al. 2024; Lardies et al. 2021). These herbivorous intertidal organisms, with differing distribution ranges and larval dispersal capabilities, provide a model to explore how environmental variability in upwelling systems affects physiological performance and survival. By combining the CMR methods with physiological measurements in populations from contrasting upwelling zones, we aimed to bridge experimental and field‐based research, shedding light on mechanisms of adaptation and survival in rapidly changing marine ecosystems.

## 2 Materials and Methods

### 2.1 Study Area and Environmental Data

This study was conducted in two geographically contrasting upwelling systems: one semi‐permanent and the other seasonal. The semi‐permanent upwelling system is located in Punta Lengua de Vaca near Talcaruca (30°43′ S, 71°41′ W; Figure 1) (Torres et al. 2011; Fernández et al. 2024), while the seasonal upwelling system is in Quintay Bay (33° 11′ S, 71° 43′ W; Figure 1) (Pulgar et al. 2011). Semi‐permanent upwelling is generally more stable, sustained year‐round by persistent equatorward winds and coastal jets, whereas seasonal upwelling depends on winds that intensify only during certain periods, producing intermittent and more variable events (Letelier et al. 2009; Bravo et al. 2016). Although these coastal areas are approximately only 300 km apart, they differ significantly in environmental parameters and variability (see below).

Sea surface temperature (SST) data were obtained from the ERA5 dataset, provided by the Copernicus Climate Change Service (C3S) (Hersbach et al. 2020; Olauson 2018). SST data for the study sites were extracted for the period January 2020 to December 2022. The regions of interest were defined using each site's geographic coordinates, with an influence radius adjusted to ERA5 spatial resolution. Data extraction and processing were performed using the *ncdf4* package in R version 4.2.3 (R Core Team 2023).

To characterize carbonate system parameters at each site, 30 discrete water samples were collected over 2 years. pH measurements on the total scale (pHT) were conducted within 60 min of collection using a Metrohm 826 pH Mobile Meter equipped with a combined double‐junction electrode. The electrode was calibrated with TRIS buffers (pH = 8.089) at 25°C in a thermo‐regulated water bath. Total alkalinity (TA) was analyzed from discrete water samples collected in 500 mL borosilicate glass bottles (Corning). Each sample was treated with 0.2 cm^3^ of 50% saturated mercuric chloride solution and sealed with Apiezon L grease for laboratory transportation. Samples were stored for no longer than 2 months under cool, dark conditions before analysis. AT was measured via automated potentiometric titration (Haraldsson et al. 1997), analyzing three to five subsamples per bottle. *p*CO_2_ and Ω for calcite and aragonite were calculated using CO2SYS software (Pierrot et al. 2006), based on averaged pH_T_, AT, and SST (Table 1).

### 2.2 Capture, Mark, and Recapture

The search for individuals of both species was conducted monthly over 26 months, from November 2019 to December 2021 (see Table 1). At each site, the search area remained consistent, covering 400 m of coastline in Talcaruca and 200 m in Quintay (within the coastal marine research station of Universidad Nacional Andrés Bello), with the search area extending across the intertidal zone (with a width of approximately 4–6 m). Although the coastline length differed between sites, capture effort was standardized by search duration, number of researchers, and intertidal width surveyed, ensuring comparability across locations. Monthly searches for live animals or shells of dead individuals were carried out at low tide by a consistent team of two researchers over 90 min, ensuring identical capture effort across sites. No major interruptions due to weather conditions occurred during the study period, minimizing potential sampling bias.

**TABLE 1:** Summary of the number of captures of individuals of *Chiton granosus* and *Scurria zebrina* carried out in the localities of Talcaruca and Quintay.

Location	Species	Nov‐19	Dec‐19	Jan‐20	Feb‐20	Mar‐20	Apr‐20	May‐20	Jun‐20	Jul‐20	Aug‐20	Sept‐20	Oct‐20	Nov‐20	Dec‐20	Jan‐21	Feb‐21	Mar‐21	Apr‐21	May‐21	Jun‐21	Jul‐21	Aug‐21	Sept‐21	Oct‐21	Nov‐21	Dec‐21
*Captures per month in the Talcaruca locality*																											
Talcaruca	*C. granosus*	42	16	21	—	—	—	—	—	—	—	—	—	—	27	35	—	—	—	25	4	21	—	—	13	29	—
	*S. zebrina*	13	—	24	—	—	—	—	—	—	—	—	—	—	12	5	—	—	—	—	2	3	—	—	—	—	—
*Captures per month in the Quintay area*																											
Quintay	*C. granosus*	37	28	20	—	31	—	12	—	—	—	—	—	—	21	27	—	27	—	28	12	36	10	—	14	—	—
	*S. zebrina*	11	29	16	—	20	—	22	—	—	—	—	—	—	19	27	—	10	—	3	17	12	2	—	—	—	—

*Note:* The sign (—) means that no search was performed in that month.

Individuals were marked during the first capture using bee tags attached to the shell with adhesive (see Aguilera and Navarrete 2007). Based on shell marks remaining after tag loss, we estimated that fewer than 5% of tags detached over time. If a deteriorated but identifiable tag was observed in the field, the individual was retagged. To construct the CMR data history, only the observed code of previously marked individuals was recorded without recapturing the animal. Captured individuals were transported to the Bioengineering Laboratory of the Universidad Adolfo Ibáñez (Santiago, Chile) for morphological and physiological measurements. On average, no more than 10 days elapsed between capture, acclimatization, measurement, and release. All individuals were returned to their original capture sites to ensure consistent handling across locations.

### 2.3 Laboratory Maintenance and Experimental Conditions

In the laboratory, individuals were maintained for 10 days under controlled conditions (Thompson et al. 2012; Manríquez et al. 2021) that simulated a semi‐diurnal tidal cycle, featuring a 12 h:12 h light–dark photoperiod, flowing seawater (~12°C), pH (8.0), and salinity (~33 psu), closely matching the annual averages for Talcaruca and Quintay (Vargas et al. 2017; Broitman et al. 2018). Both species were fed with *Ulva* spp. three times a week.

Feeding was suspended 24 h before physiological measurements (i.e., metabolic rate and heart rate), to standardize conditions and eliminate variability due to digestion. Animals also rested for at least 24 h between metabolic and heart rate measurements, and the order of experimental procedures was randomized to prevent temporal effects.

### 2.4 Metabolic Rate Measurements

Standard metabolic rate (SMR) was measured as oxygen consumption (mgO_2_ h^−1^ g^−1^) using fiber optic oxygen sensors (Precision Sensing, GmbH, Germany) connected to a PreSens Oxy‐4 mini respirometer. Each individual was placed in a 1 L glass respirometry chamber filled with UV‐filtered water, maintained at 14°C using a recirculating water bath (BOYU, Model L075). Sensors were calibrated before measurements using saturated Na_2_SO_3_ (0% oxygen) and aerated seawater (100% oxygen) following (see Gaitán‐Espitia et al. 2014; Rodríguez‐Romero et al. 2022). Oxygen levels were recorded every 15 s for 60 min. The first and last 5 min of each recording were discarded to minimize artifacts related to handling stress. The average oxygen consumption during the intermediate 50 min was considered the SMR of each individual. Control measurements, using empty respirometry chambers under identical conditions, confirmed that oxygen reduction never exceeded 3% during the experimental period.

### 2.5 Heart Rate Measurements

Heart rate was measured by immobilizing individuals on a humid plate using adhesive tape and placing them in a thermoregulated bath (JeioTech, model RW‐2025) maintained at 14°C. Animals were then transferred to plastic chambers with six compartments (200 × 200 × 100 mm) filled with seawater and exposed to the same temperature for 30 min. Heart rate was measured with an AMP 03 heartbeat amplifier (Newshift Lda) connected to an oscilloscope, with results expressed in beats per minute. Measurements were performed at a consistent time of day to minimize circadian or tidal rhythm effects on cardiac activity. Electrical signals were recorded as electrocardiograms using the Multi Channel Oscilloscope v1.31.2.0 software (TiePie engineering) (Rodríguez‐Romero et al. 2022), and the mean heart rate was calculated for each individual at 14°C.

### 2.6 Morphological Variables

To assess growth rate and morphological changes, the length of each individual was measured using an electronic caliper (Mitutoyo, Sakado, Japan). Buoyant weight, a proxy for calcification and growth rates (Lardies et al. 2017; Palmer 1982), was measured at the beginning and end of the laboratory period. Weights were obtained using an analytical balance (±0.1 mg, AUX 220, Shimadzu, Kyoto, Japan) with the entire animal submerged in seawater. Initial length and weight measurements were recorded after the acclimatization period but before metabolic rate measurements.

### 2.7 Statistical Analysis I: Physiology and Morphology

To analyze physiological performance, we used linear mixed models with restricted maximum likelihood estimation. The effects of locality, buoyant weight, and their interaction on metabolic rate and heart rate were evaluated, with the capture date included as a random factor. Additionally, the effects of locality and date of capture date on buoyant weight were also evaluated. Metabolic rate and buoyant weight were log‐transformed (base 10) to meet normality assumptions. *p* values for fixed effects were calculated using the package lmerTest (Kuznetsova et al. 2017) with type III sums of squares based on the Satterthwaite approximation for the denominator degrees of freedom. For each model, we report effect sizes with 95% confidence intervals in addition to *p*‐values. Model selection was based on parsimonious fit, using likelihood ratio tests (anova function in lme4) and AIC comparisons (MuMIn package), retaining the most parsimonious models. Statistical analyses were conducted in R version 4.2.3 (R Core Team 2023), using the lme4 package (Bates et al. 2015). Finally, we performed a one‐way multivariate analysis of variance (MANOVA) using Wilk's *λ* as a statistical test and using Dunnett *C* as a post hoc test, including all carbonate system parameters to estimate differences in environmental conditions between sites.

### 2.8 Statistical Analysis II: Survival Probability

The apparent survival probability (*ø*) for each species and study site was estimated using the Cormack–Jolly–Seber (CJS) model (Williams et al. 2002). This model defines survival as apparent because it does not differentiate between true survival and permanent emigration (i.e., dispersal) from the study site (Williams et al. 2002). However, given the life stages and species studied (i.e., postlarval stages), permanent emigration is likely negligible. Previous studies have shown strong site fidelity and limited dispersal in intertidal chitons and limpets, with individuals remaining attached to specific microhabitats for extended periods (Aguilera 2011; Montecinos et al. 2020). Therefore, we expect apparent survival to closely approximate true survival. Tag loss was < 5% and did not affect data integrity; individuals with deteriorated but identifiable tags were retagged, and missing observations were recorded as missing events in CMR histories.

The CJS model was fitted to data within a Bayesian framework using JAGS (Plummer 2003) through R's package jagsUI (Kellner 2015). To reduce the possibility of finding spurious results, we built a limited number of models for each species (Table 2), considering the following hypotheses:

1. Within a species, *ø* is constant over time, but varies with upwelling type (i.e., locality), individual size, and/or physiological performance;
2. Within a species and locality, *ø* varies over time.

**TABLE 2:** Structure of the different variations of the Cormack–Jolly–Seber model used to estimate the monthly probability of apparent survival in *Chiton granosus* and *Scurria zebrina* at the Talcaruca and Quintay localities.

Models	Explanation of the model
*ø*(*t*) *p*(*t*)	The apparent monthly survival of individuals in each location varies during each month
*ø*(*Po*) *p*(*t*)	Apparent monthly survival depends on location
*ø*(*F*) *p*(*t*)	Apparent monthly survival of individuals varies according to physiological performance
*ø*(*L*) *p*(*t*)	Apparent monthly survival of individuals varies according to body length
*ø* (*·*) *p*(*t*)	Apparent monthly survival of individuals is constant during the study period

*Note:* All models were species‐specific. The encounter probability (*p*) was always modeled with full temporal variation (*p*(*t*)), to accommodate the irregular temporal structure of our capture occasions. The covariates: Population or locality (*Po*), physiological performance (*F*), and size (*L*) were taken into account. Following classical CMR nomenclature, full temporal variation is denoted as (*t*) and constancy over time as (·).

Due to the irregular temporal structure of the data (i.e., searches were not conducted during the same months across sites and species; see Table S1), we modeled recapture probability (*p*) with full temporal variation in all model variations. Because size and physiological performance were only measured after the first capture and can vary significantly over time, we limited the dataset used for the analysis of these variables to a 6‐month CMR window. For months with no field searches, *p* was fixed at 0 in the Bayesian model. All models were fitted separately for each species and locality.

To compare differences in *ø* between species and sites, we used the posterior distribution of the parameter under the constant *ø* model, following Kéry (2010). Uninformative priors were assigned to all parameters (Kéry and Schaub 2011). Bayesian analysis was conducted using three Markov Chain Monte Carlo (MCMC) chains of 10,000 iterations each and a burn‐in of 1000. Convergence was assessed using the Gelman‐Rubin *R*‐hat statistic (i.e., *R*‐hat values < 1.1) and visual inspection of the chains (Kéry and Schaub 2011).

## 3 Results

### 3.1 Environmental Data and Capture of Mollusks

**FIGURE 2:**
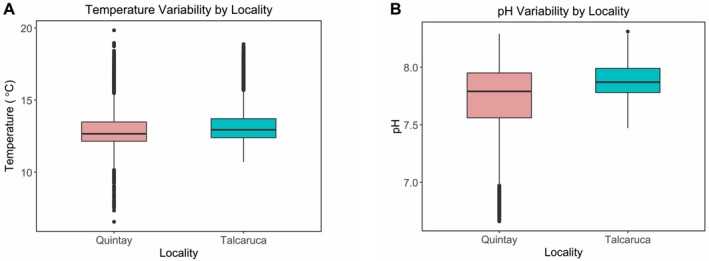
Variability of temperature (A) and pH (B) between the study localities. Boxes represent the interquartile range (IQR), with the center line indicating the median. Whiskers show the data range (excluding outliers), and dots represent outliers.

**FIGURE 3:**
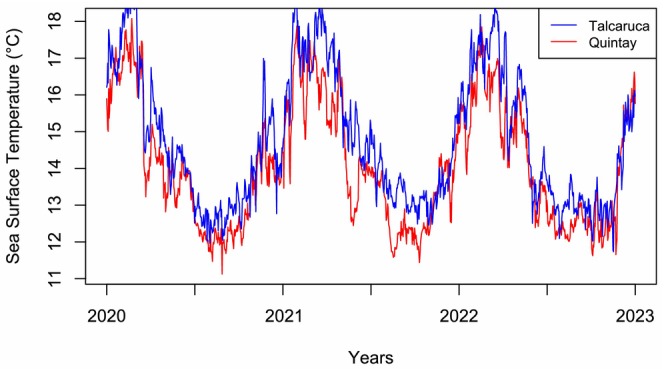
Time series of SST at Talcaruca (red) and Quintay (blue) localities for the period January 2020 to December 2022 using satellite data obtained from the ERA5 dataset, provided by the C3S.

Temperature and pH data revealed significant variability differences between localities (Figure 2, Table 3). The SST time series showed consistently lower temperatures in Quintay compared to Talcaruca during the analyzed period (2020–2022). Both sites exhibited clear seasonal patterns, with lower temperatures in winter and higher temperatures in summer (Figure 3). However, thermal peaks were more pronounced in Talcaruca, indicating greater thermal variability. Statistical analyses, including the Fligner‐Killeen test (*p* = 0.0082) and Levene's test (*p* = 0.0074), confirmed significant differences in temperature variance between the two localities.

**TABLE 3:** Summary of salinity and seawater carbonate chemistry variables measured in two study locations.

Parameter	Locality	
Upwelling regime	30° 29′ S Talcaruca	33° 13′ S Quintay
	Semi‐permanent	Seasonal
Salinity	33.53 ± 1.63	33.34 ± 2.37
pH_NBS_	8.04 ± 0.46	7.99 ± 0.24
TA (μmol kg^−1^)	2273.8 ± 37.01	2171.92 ± 225.75
CO_3_^−2^ (μmol kg^−1^)	103.2 ± 39.6	145.3 ± 34.8
*p*CO_2_ (μatm)	554.5 ± 93.4	487.65 ± 108.8
Ω_calcite_	2.69 ± 0.35	3.06 ± 0.69
Ω_aragonite_	2.04 ± 0.42	1.96 ± 0.54
SST (°C)	13.20 (8.02, 16.8)	13.04 (8.09, 17.6)

*Note:* Mean ± SD during 2019 and 2021. Temperature information includes the annual average of the SST during the same periods. The minimum and maximum temperatures are shown in parentheses. Other parameters are: Total alkalinity (TA), carbonate (CO_3_^2−^), the partial pressure of CO_2_ (*p*CO_2_), saturation states for aragonite Ω_Aragonite_, and saturation states for calcite Ω_Calcite_.

These patterns were further supported by in situ temperature and pH measurements, which revealed significant differences in variability between localities. Temperature variance differed significantly according to the Fligner–Killeen test (*p* = 0.002) and Levene's test (*p* < 0.001), as did pH variance (Fligner–Killeen test: *p* < 0.001; Levene's test: *p* < 0.001). Although mean values of most environmental parameters were comparable between Talcaruca and Quintay, variability was markedly higher in Talcaruca for parameters such as pH, TA, and CO₃^2−^ as indicated by substantially greater standard deviations (Table 3). Multivariate analysis of carbonate system parameters showed large contrasts between sites (Wilk's *λ* = 0.085; *F*‐exact test = 12.192; *p* < 0.001). However, both Dunnet's C as post hoc test for MANOVA and univariate ANOVAs of each environmental parameter did not show differences between sites.

A total of 783 adult individuals were captured: 536 of *C. granosus* (Talcaruca: *N* = 233, Quintay: *N* = 303) and 247 of *S. zebrina* (Talcaruca: *N* = 59, Quintay: *N* = 188) (see Table S1). Median captures per sampling event in the semi‐permanent upwelling locality (Talcaruca) were 21 for *C. granosus* and 9.5 for *S. zebrina* , while in the seasonal upwelling locality (Quintay), median captures were higher at 28 for *C. granosus* and 21.5 for *S. zebrina* .

### 3.2 Population Differences in Physiology and Morphology

**FIGURE 4:**
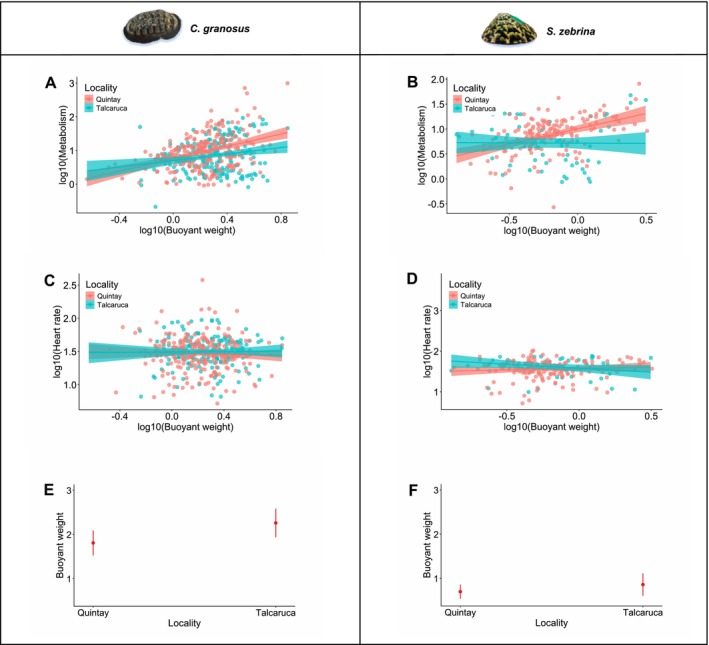
Relationship between metabolism (mgO_2_ h g^−1^) and buoyant weight (mg) in each locality of *Chiton granosus* (A) and *Scurria zebrina* (B) (red = Quintay; blue = Talcaruca). Relationship between heart rate (beats min^−1^) and weight (mg) in *Chiton granosus* (C) and *Scurria zebrina* (D) The shaded area represents 95% confidence intervals. The mean (dots) and standard deviation (bars) of buoyant weight (mg) at each location studied for *Chiton granosus* (E) and *Scurria zebrina* (F) are shown.

The mixed effects model evaluating the effects of locality, buoyant weight (weight), and their interaction on metabolic rate and heart rate revealed notable differences between the studied species. For *C. granosus*, neither the interaction between locality and buoyant weight (*F*_1,465.58_ = 1.61, *p* = 0.2) nor locality alone (*F*_1,24.60_ = 0.06, *p* = 0.8) significantly influenced metabolic rate. However, buoyant weight showed a strong and positive effect on metabolism (*F*_1,465.58_ = 40.75, *p* < 0.001, Figure 4A). In contrast, for *S. zebrina* , the interaction between locality and buoyant weight significantly affected metabolic rate (*F*_1,221.74_ = 11.93, *p* = 0.007). In particular, both intercepts and slopes differed between localities (Figure 4B), indicating variations in energy expenditure associated with buoyant weight. Additionally, the differences in intercepts suggest differences in metabolism in a general way, without the influence of buoyant weight.

Regarding heart rate, no significant effects of the locality * buoyant weight interaction (*F*_1,394_ = 0.05, *p* = 0.81), locality alone (*F*_1,33_ = 0.55, *p* = 0.46), or buoyant weight (*F*_1,394_ = 0.15, *p* = 0.7) were observed in *C. granosus* (Figure 4C). Similarly, in *S. zebrina* , heart rate was not significantly affected by the interaction (*F*_1,18_ = 0.01, *p* = 0.06), locality (*F*_1,18_ = 0.01, *p* = 0.91), or buoyant weight (*F*_1,130_ = 0.747, *p* = 0.389) (Figure 4D). Finally, the influence of locality on buoyant weight was not statistically significant for *C. granosus* (*F*_1,20.07_ = 4.26, *p* = 0.052) and for *S. zebrina* (*F*_1,19.8_ = 1.1, *p* = 0.3) (Figure 4E,F).

### 3.3 Capture–Recapture

**FIGURE 5:**
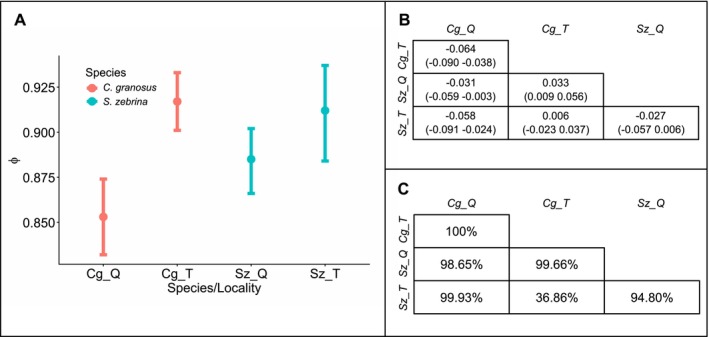
Shown are the mean (dots) and 95% Bayesian credible intervals (bars) for the posterior distribution of the probability of apparent survival (*ø*) obtained from the Cormack‐Jolly‐Seber model with structure *ø*(*P*) *p*(*t*) (A) showing the effect of locality on survival (red = *Chiton granosus*; blue = *Scurria zebrina*). *Chiton granosus* in Quintay (Cg_Q), *Chiton granosus* in Talcaruca (Cg_T), *Scurria zebrina* in Quintay (Sz_Q), and *Scurria zebrina* in Talcaruca (Sz_T). In addition, (B) shows a pairwise comparison of the mean of the estimated parameter between populations or Bayesian effect estimates (Kéry 2010) and the probability of differences in mean survival between two populations (C).

Models comparing survival probabilities between localities with constant survival over time (ø(·)) and locality‐dependent survival (ø(Po)) revealed significant differences between semi‐permanent (Talcaruca) and seasonal (Quintay) upwelling sites for both species. The probability of a difference in ø(·) between Talcaruca and Quintay was 100% in *C. granosus* and 94.8% in *S. zebrina* (Figure 5C). The effect size, representing the magnitude of differences in ø(·) between localities, was larger in *C. granosus* compared to *S. zebrina* (Figure 5B). Annual survival probabilities, derived from monthly estimates, were higher in Talcaruca than in Quintay for both species: 0.36 vs. 0.15 in *C. granosus* (2.4 times higher) and 0.33 vs. 0.23 in *S. zebrina* (1.43 times higher).

**FIGURE 6:**
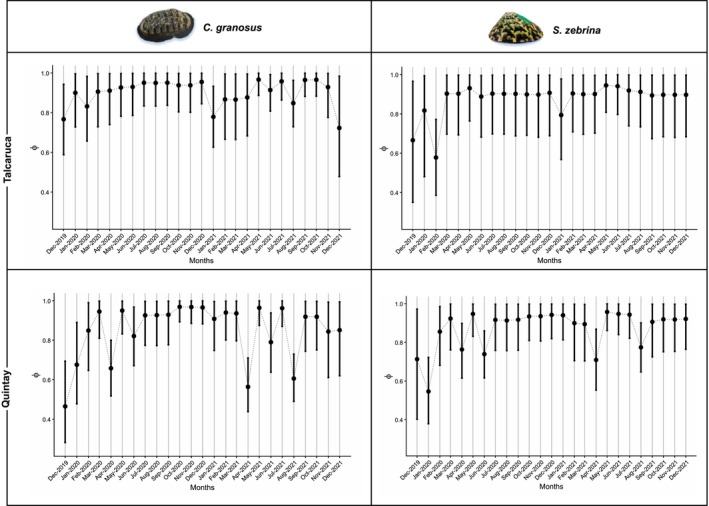
Relationship between body length and survival probability (A) and the relationship between physiological yield and survival probability (B) in *Chiton granosus* and *Scurria zebrina* species at Talcaruca and Quintay localities. Mean (dots) and 95% Bayesian credible intervals (bars) for parameter *β* of the *ø*(*L*) *p*(*t*) model (A) and the *ø*(*F*) *p*(*t*) model (B) at Talcacura (blue) and Quintay (red) are shown. The parameter *β* presents the slope of a linear regression with the predictor body length (A)/physiological performance (B) and the logit of the survival probability as the response variable.

Temporal variation in survival was also evident. The *ø*(*t*) *p*(*t*) model showed greater survival variability at the seasonal upwelling locality (Quintay) for both species, with notable decreases in survival during December 2019, January, April, and June 2020, as well as April and August 2021. In contrast, survival was more stable over time in Talcaruca for both species (Figure 6).

**FIGURE 7:**
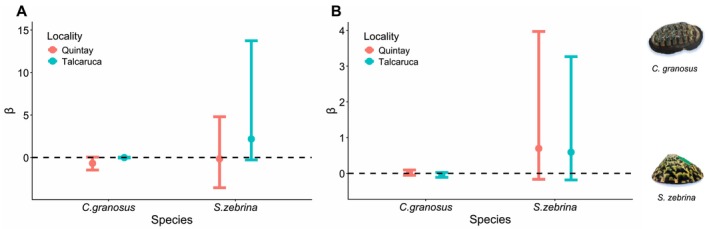
Monthly apparent survival probability at Talcacura and Quintay in populations of *Chiton granosus* (A, C) and *Scurria zebrina* (B, D). Shown are the mean (dots) and 95% Bayesian credible intervals (bars) for the posterior distribution of the probability of apparent survival (*ø*) obtained from the Cormack‐Jolly‐Seber model with structure *ø* (*t*) *p*(*t*).

The slope (*β*) of the model *ø*(*L*) *p*(*t*) showed no significant relationship between body length and survival probability in either species. For *C. granosus* , the mean *β* value was close to zero (Figure 7A), while in *S. zebrina* , the mean *β* was closer to 1, but the credible interval included 0 (Figure 7A), indicating that the evidence for a positive relationship is inconclusive. Similarly, no significant relationship was found between metabolic rate and survival probability (model *ø*(*F*) *p*(*t*); Figure 7B). In both *C. granosus* populations and the *S. zebrina* population from the seasonal upwelling locality (Quintay), the average *β* value was near zero. In the *S. zebrina* population from the semi‐permanent upwelling locality (Talcaruca), the *β* value was close to 1; however, the credible interval overlapped zero, meaning the effect cannot be considered statistically supported.

## 4 Discussion

Heterogeneous environments drive phenotypic plasticity and/or local adaptation in organisms (Bonamour et al. 2019). Understanding how marine organisms respond to these conditions is critical under ongoing global change. This study assessed the physiological, morphological, and survival responses of two intertidal mollusk species (*Scurria zebrina* and *Chiton granosus*) in contrasting upwelling regimes using field data and laboratory measurements. Our results show: (1) No differences in heart rate between populations of either species; (2) Higher metabolic rate in *S. zebrina* at the seasonal upwelling site (Quintay); (3) Similar calcification rates for both species across locations; and (4) Higher survival at the semi‐permanent upwelling site (Talcaruca) for both species.

### 4.1 Physiological Responses to Upwelling

We observed no significant differences in heart rate between populations of either species, suggesting physiological stability in this parameter under contrasting upwelling regimes. However, *S. zebrina* exhibited a higher metabolic rate at the seasonal upwelling site (Quintay) coupled with a positive relationship between metabolism and calcification (buoyant weight). This indicates that shell formation is more energetically costly at this site, likely due to lower pH and higher shell dissolution rates (see Lagos et al. 2016). In contrast, the flat metabolic slope at the semi‐permanent upwelling site (Talcaruca) suggests physiological tolerance to shell dissolution, possibly facilitated by greater plasticity (Lardies et al. 2021).

For *C. granosus* the relationship between metabolism and calcification was consistent across locations, suggesting limited adaptive differentiation to different upwelling regimes, likely due to higher dispersal ability and gene flow (see Räsänen and Hendry 2008). These findings align with studies reporting species‐specific responses to low pH, where calcification rates may remain stable or increase under certain conditions (Wood et al. 2008; Ries et al. 2009; Lagos et al. 2016). Moreover, recently Fernández et al. (2024) showed that *C. granosus* from the Talcaruca upwelling zone represents a local population with wide tolerance ranges that may be capable of withstanding future upwelling intensification in the Southern Eastern Pacific.

### 4.2 Effects of Upwelling on Survival

**FIGURE 8:**
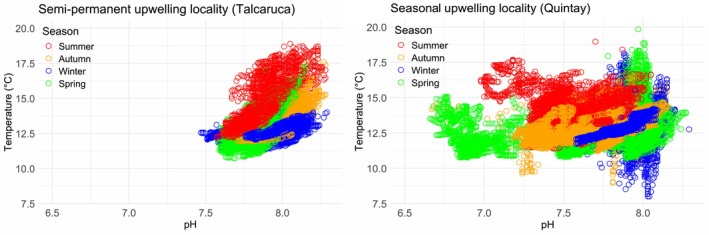
Relationship between temperature (°C) and sea pH at each location, Talcaruca and Quintay, separated by seasons (red = summer; blue = winter; orange = autumn; green = spring).

Studies estimating the survival of invertebrates in the wild are scarce, and to our knowledge no comparable long‐term survival estimates exist for marine mollusks. Therefore, we briefly refer to examples from freshwater and terrestrial gastropods to illustrate the range of survival probabilities reported in invertebrates more broadly. Among the few existing studies on mollusks, Villella et al. (2004) recorded an annual survival rate exceeding 90% for four different species of freshwater mussels, and Miranda and Fontenelle (2015) reported a 96.7% annual survival probability for a terrestrial gastropod. On the other hand, Ryser et al. (2011) compared the survival rates of two terrestrial gastropod species, one invasive, and found that the survival rate of the invasive species was significantly higher, reaching approximately 78% annual survival probability, while the displaced species barely reached 14%. Similarly, Nakamura et al. (2023) identified survival issues in a freshwater mussel that experienced a constant annual decline, reaching 14% annual survival in 2020. These examples of species with low survival rates, which raise alarms among researchers, have values similar to those found for *C. granosus* in the seasonal upwelling locality. Survival rates were significantly higher at the semi‐permanent upwelling site (Talcaruca) for both species, with annual survival probabilities 2.4 times higher for *C. granosus* and 1.43 times higher for *S. zebrina* compared to the seasonal upwelling site (Quintay). These results reflect more stable environmental conditions at Talcaruca, whereas Quintay experiences frequent low‐pH events, which are known to be highly corrosive for shelled mollusks (Bogan et al. 2019; Dorey et al. 2022). Seasonal upwelling sites like Quintay likely impose higher energetic demands on mollusks, increasing mortality, particularly during spring and summer when upwelling intensity peaks (see Figure 8).

*S. zebrina* showed higher survival than *C. granosus* at the seasonal upwelling site (Quintay). This could be attributed to its effective behavioral thermoregulation and phenotypic plasticity in metabolism (Broitman et al. 2018; Manríquez et al. 2021). Genetic analyses indicate high diversity and signals of divergent selection in *S. zebrina* populations near our study sites (Saenz‐Agudelo et al. 2022), potentially contributing to its resilience under variable conditions. However, genetic homogeneity between populations suggests local adaptation is unlikely (Saenz‐Agudelo et al. 2025).

Our findings highlight the vulnerability of intertidal mollusks to environmental stressors associated with upwelling. Of the 59 days with pH below 7.3 recorded at Quintay, 49.2% occurred in spring, 32.2% in autumn, and 18.6% in summer (Figure 8). The months of lower survival correspond mainly to summer and autumn, likely due to the cumulative effects of strong upwelling events in spring and milder events in summer and autumn (Dorey et al. 2022).

Projected intensification of upwelling in the Southeastern Pacific (SEP) due to global warming is expected to exacerbate these stressors, with colder, more acidic waters negatively impacting calcification and survival (García‐Reyes et al. 2015; Rykaczewski et al. 2015; Sydeman et al. 2014). Mollusk species with broad geographical ranges and high phenotypic plasticity, such as *S. zebrina* , are likely to fare better under these conditions (Rodríguez‐Romero et al. 2022).

The proximity of Talcaruca to the distributional limit of *S. zebrina* (a biogeographic break zone) likely contributes to greater phenotypic plasticity in this population (Barria et al. 2014; Broitman et al. 2018). This zone, characterized by abrupt environmental variability, may drive adaptive changes in phenotypic traits. Similarly, the combination of genetic diversity and environmental variability at Quintay highlights the importance of understanding local dynamics to predict species' responses to future changes.

This study underscores the critical role of upwelling intensity and variability in shaping the physiological and survival responses of intertidal mollusks. Although *C. granosus* appears adapted to environmental variability, reflected in a higher survival probability in semi‐permanent upwelling, *S. zebrina* demonstrates a greater capacity for resilience through metabolic plasticity. Our findings emphasize the importance of incorporating regional environmental variability into conservation and management strategies for marine species under global change.

## Author Contributions

**Alejandro Hernández‐Dauval:** conceptualization (equal), methodology (equal), software (equal), writing – original draft (equal), writing – review and editing (equal). **Andrés Valenzuela‐Sánchez:** formal analysis (equal), software (equal), writing – original draft (equal), writing – review and editing (equal). **Marco A. Lardies:** conceptualization (equal), funding acquisition (equal), investigation (equal), methodology (equal), supervision (equal), writing – review and editing (equal). **Leonardo D. Bacigalupe:** conceptualization (equal), formal analysis (equal), methodology (equal), software (equal), writing – original draft (equal), writing – review and editing (equal).

## Funding

This work was funded by ANID FONDECYT N° 1240367 and PIA ANID ANILLOS ACT240004.

## Ethics Statement

All procedures were conducted in accordance with the Research Ethics guidelines of ANID‐Chile (2019) and were approved by the Bioethics Committee of Adolfo Ibáñez University (No. 20/2019).

## Consent

The authors have nothing to report.

## Conflicts of Interest

The authors declare no conflicts of interest.

## Data Availability

All data and code underlying the results presented in this manuscript are publicly available in the Dryad Digital Repository: https://doi.org/10.5061/dryad.rfj6q57pb.
